# A Dive into Delivery of Oral Hygiene Advice Based on the Personalized Oral Hygiene Advice Model

**DOI:** 10.7759/cureus.56606

**Published:** 2024-03-20

**Authors:** Nusaibah S Nordin, Haslina Taib

**Affiliations:** 1 Unit of Periodontics, School of Dental Sciences, Universiti Sains Malaysia, Kota Bharu, MYS

**Keywords:** oral care, oral health professionals, customized oral hygiene advice, personalized dentistry, oral health

## Abstract

Oral health's impact on overall well-being highlights the importance of preventive measures through effective oral hygiene practices. Currently, there is growing recognition of the need for customized oral hygiene advice depending on the patient’s unique needs and circumstances. This narrative review addresses the gap in understanding the significance of personalized guidance through the proposal of the Personalized Oral Hygiene Advice Model (POHAM) as a comprehensive guide for oral health professionals. This model was developed to adapt to evolving patient demographics and diverse challenges, promoting a patient-centric and effective oral health approach. The POHAM comprises a flow chart of strategies from establishing a good rapport with patients, conducting comprehensive assessment through history-taking, psychosocial and technology proficiency evaluation, tailored education modules, and customized oral care product recommendations until the reassessment. These strategies aim to enhance patient engagement and adherence, as well as act as a guide for oral health professionals to use in the clinical setting before and during the course of oral treatment. Nevertheless, continued research, education, and technological advancements are needed to realize the full potential of personalized oral hygiene strategies, ensuring a transformative and sustainable oral healthcare landscape.

## Introduction and background

Introduction

Oral health plays a vital role in an individual’s overall well-being, influencing not only physical health but also psychological and social aspects of life. Preventing oral diseases through effective oral hygiene practices is a cornerstone of dental care [1,2]. As dentistry evolves, there is a growing recognition of the importance of tailoring oral hygiene advice (OHA) according to each patient's unique needs and circumstances. Despite an exhaustive list of information available on oral health, a gap exists in understanding the importance of providing personalized oral hygiene guidance. Standardized recommendations may not sufficiently address the variations in local and systemic factors influencing an individual’s oral health [3]. This narrative review highlights the importance of tailoring oral hygiene advice (OHA) to individual needs and circumstances, acknowledging the gap in understanding the significance of personalized guidance amidst the abundance of oral health information.

Oral health-related risk factors

Local and systemic risk factors significantly impact the development and progression of oral diseases [4]. Local risk factors can be divided into acquired or anatomical factors. The existence of plaque, calculus, overhanging restorative margins, and poorly contoured restorations are part of acquired local factors that may increase the risk of developing periodontal disease and caries. The anatomical local factors include the presence of enamel pearls, concavities, crowding of teeth, and furcation involvement. Systemic factors encompass variables, such as systemic diseases, socioeconomic status, genetics, age, and smoking [5]. These factors may affect oral health, particularly periodontium, in a generalized manner. Hence, it is important to address an individual need in terms of oral hygiene care, behavior, and lifestyle changes by carefully analyzing the risk factors and adopting a rather holistic approach to maintain good oral health.

The shift toward personalized healthcare

Current practices in the medical and dental fields have slowly shifted towards personalized care. Personalized medicine is the practice of using patients’ information with regard to the person’s genetic predisposition, proteins, and environment to prevent, diagnose, and treat any diseases [6,7]. Following the same theme, this form of medicine can be extended to dentistry in terms of oral health, which is considered as an essential part of overall well-being [8].

It is crucial that personalized oral hygiene advice (OHA) is integrated as part of the prevention stage in oral health therapy. In 2020, the European Federation of Periodontology (EFP) came out with a clinical practice guideline (CPG) for the treatment of periodontitis. They highlighted the importance of a personalized care plan that consisted of the delivery of adequate information to patients concerning their diagnosis, etiology, and causes of the diseases, risk factors involved, treatment options available, as well as risks and benefits of the treatment [9]. This plan can be modified along the treatment progress depending on the circumstances and changes in their oral and general health. All the information should be delivered in the first stage of the stepwise treatment approach recommended by the CPG, emphasizing guiding behavior change in terms of the removal of supragingival dental plaque such as toothbrushing, flossing, interdental cleaning and management of the risk factors involved.

The need for personalized oral hygiene advice

Despite the need for personalized OHA for each individual due to variations in the risk factors associated with the person, the literature lacks guidelines for oral health practitioners to apply this approach to their patients. Oral health professionals, such as dental specialists, general dental practitioners, dental hygienists, and other allied dental practitioners, are increasingly confronted with a diverse population of patients while presenting unique challenges and requirements individually. This situation called for a specific approach in delivering oral hygiene instructions to them, as emerging evidence has suggested that personalized OHA may enhance patient compliance, thereby contributing to better oral health outcomes [10]. Hence, this review aims to improve the personalization aspect of OHA delivery by offering a comprehensive guide to oral health professionals through the Personalized Oral Hygiene Advice Model (POHAM), as a one-size-fits-all approach is no longer relevant and may not yield optimal results to the patients.

## Review

Factors influencing patients’ adherence to oral hygiene advice

Ensuring patients’ adherence to OHA is integral to the success of personalized care interventions. Before implementing a personalized oral hygiene care approach, oral health care practitioners should have an overview of the factors that may influence patients’ adherence to OHA so that it can facilitate them in delivering optimum oral hygiene advice to the patients.

According to a study on the factors influencing adherence to oral hygiene care, several factors were identified from focus group discussions conducted among periodontal patients [11]. The factors include inadequate knowledge of oral care from the patients, their behavior and past neglect concerning oral hygiene care, the relationship between dentists and patients, their cultural background and beliefs, as well as cost factors involving oral hygiene cleaning aids [12]. Another study came out with a model for patient compliance, and it was suggested that the relationship between patients and periodontists has a direct association with compliance [13]. The important characteristics of the clinician, specifically periodontists, include their communication and clinical skills, as well as good patient management. In terms of patient factors, oral health awareness, satisfaction, and experience from prior treatment all had a positive impact on their compliance. Other variables that may also influence their compliance with oral care include factors such as duration and complexity of treatment, age, gender, level of education, personality traits, and family and/or peer influence. Understanding these multifactorial challenges enables oral health professionals to develop personalized recommendations that align with the patient’s unique circumstances, thereby promoting sustainable adherence to oral hygiene advice delivered by their clinician. 

Strategies for personalized oral hygiene advice

**Figure 1:**
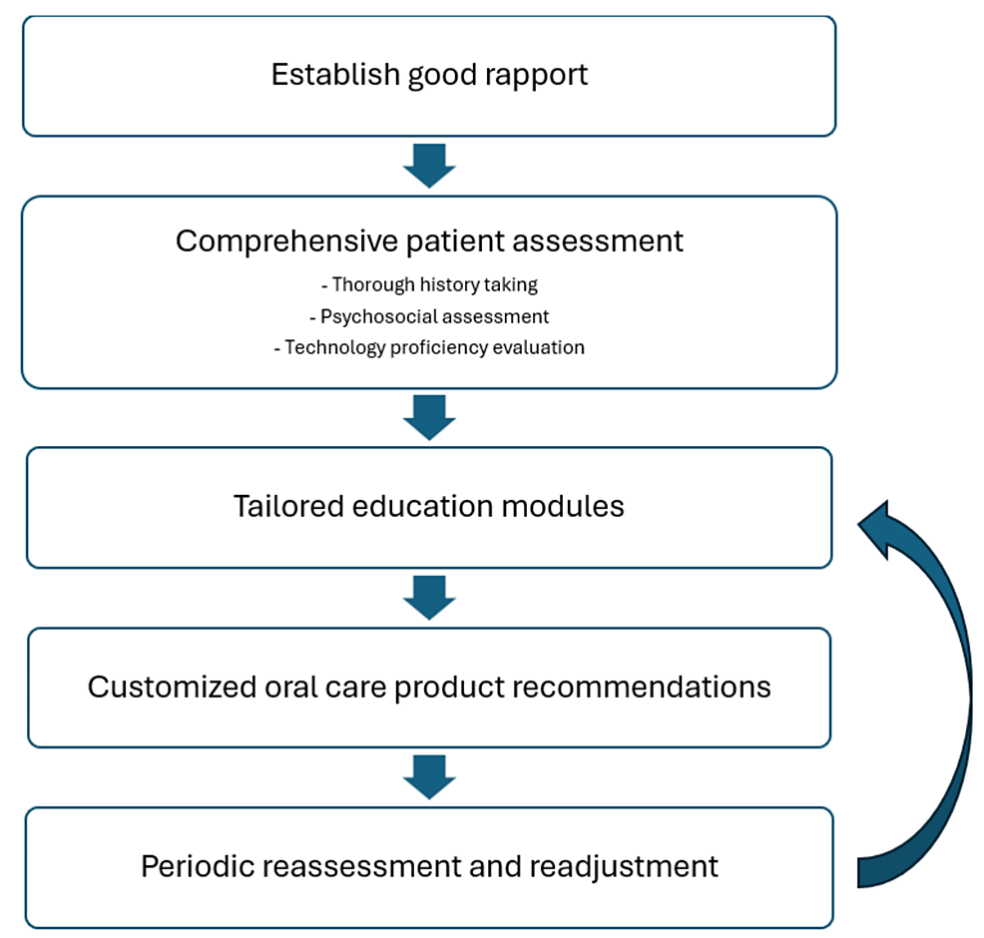
Proposed model of strategies for POHAM by oral health professionals. POHAM: Personalized Oral Hygiene Advice Model. Note: This image is the author's own creation.

In response to the evolving field of dentistry, with the oral health prevention area in particular, we proposed POHAM for oral health professionals to adopt concerning the delivery of personalized oral health advice to patients (Figure 1).

Establish Good Rapport Through Effective Communication Skills

The first strategy is to establish a good rapport with the patient. Studies have suggested that the relationship between dentists and patients is vital to increasing patients’ adherence to OHA [11,13]. The building of mutual trust and clear communication between the clinician and patients plays a pivotal role in fostering comprehension and motivation [14] because, at the early stage of therapy, it is not uncommon for the patients to be anxious and uncertain about upcoming procedures [15]. The role of oral health professionals encompasses not only the prevention and treatment of the disease but also making sure that patients understand the causes and risk factors involved in the disease [9]. This can only be achieved by having essential skills in communication such as listening well, the ability to use easy and non-jargon words, being able to comfort and reassure patients with dental anxiety, and managing to deliver and promote behavior changes [16]. Clinicians can start a small talk with the patients before embarking on full dentist mode to avoid a threatening environment, as sometimes the mere presence of the dental office can trigger anxiety in the patients [15]. This approach will allow them to become slowly comfortable and willing to share the details of their related information, thereby facilitating the development of customized oral health therapy in general and OHA in particular for the patients.

Comprehensive Patient Assessment

Prior to formulating a personalized care plan for a patient, it is imperative to conduct a comprehensive assessment of their overall risk profile, encompassing personal details, medical and dental histories, social and lifestyle factors, dietary habits, socioeconomic status, cultural beliefs, and anxiety levels [17]. This assessment commences upon the patient’s arrival at the dental clinic and continues into the surgical room, involving the evaluation of their gaits, physical capabilities, emotional state, and whether they are accompanied or alone. Additionally, when the patient is engaging in informal conversations with dental staff and oral health professionals, it will allow the evaluation of the patient’s ability to comprehend verbal information and assess their expectations regarding the visit and dental treatment in general [16]. Such an approach facilitates the delivery of varying levels of care and support, recognizing that different demographic groups may necessitate distinct approaches for oral health assessment and treatment planning. 

As part of patient evaluation, psychosocial assessment is also important to address. Psychosocial is a term that comprises the states of an individual's mental, social, emotional, and spiritual health [18]. Recognizing the impact of emotional and psychological factors allows oral health professionals to implement strategies that resonate with individual patients, ultimately enhancing their adherence to personalized OHA. Anxiety can have some effects on the cognitive functions of a person [19,20]. In this condition, patients may not have the ability to concentrate and digest the information delivered by their oral health professionals, depending on whether they are in ‘high fear’ or ‘low fear’ groups [21]. The OHA delivered without recognizing and addressing the patient’s psychosocial state might be ineffective as they cannot absorb and retain the much-needed information. Studies found that patients often fail to remember correctly the information delivered by their healthcare practitioners, up to 40-80% of the time [22,23]. Nevertheless, oral health professionals can try the approach of gradual education. The OHA should be broken down into small and manageable steps. Information should be provided gradually, allowing patients to process and ask questions at their own pace so that they can retain as much information as possible per visit. It is pertinent to provide positive reinforcement to the patients, as it helps to build their confidence and reduce anxiety about oral care [24]. Dental professionals should acknowledge the patient’s feelings [25], respect their autonomy while simultaneously involving them in the decision-making regarding their oral health, as well as allow them to set the pace and take breaks if needed.

Another important factor that needs to be evaluated is technology proficiency evaluation. It is without doubt that the world has become more advanced in terms of technology and artificial intelligence, and this does not exclude the dental field [26]. Depending on the patient's demographic status, their proficiency in the technology might differ. By evaluating this area, oral health professionals can interact with patients accordingly and guide them on how to use technology to empower themselves by taking an active role in their oral health care [27]. The use of smartphones, apps, online platforms, and digital tools such as smart toothbrushes [28] or virtual reality [29] can provide personalized oral hygiene plans through telehealth, remote monitoring, reminders, and access to educational resources that can be used by the patients to reach at their convenience [30].

Tailored Education Modules

All the information extracted from the patients should be carefully evaluated because each factor can become part of the contributing/risk factor for developing any oral diseases, with periodontal diseases and caries being the most common oral health problems [31]. For example, age and physical abilities are some of the factors that are critical for oral health professionals to be mindful of, due to the differences in the formulation of OHA. Even in the same age groups, patients with weak or paralyzed limbs due to a history of stroke need a specific and customized OHA that suits their conditions [32]. Commonly, the instructions to the elderly groups might have more emphasis on the hygiene of their remaining teeth, prevention of periodontal diseases, and care of the prosthesis inside the oral cavity, while OHA for toddlers and preschoolers should be focused on taking care of their deciduous teeth and the importance of erupting permanent dentition. These differences called for customization of OHA in terms of words to be used, the way of delivering the information, and different product recommendations. Another factor that should also be highlighted is the socioeconomic factor. Patients with financial difficulties might not be able to afford various dental cleaning aids in the market even though they need them to improve their oral hygiene [12]. Hence, oral health professionals must acknowledge this issue and try to accommodate the patients by formulating a unique OHA depending on their circumstances and attempting to modify the current oral hygiene technique instead of hastily introducing various dental cleaning aids to them [33]. Tailoring advice to align with patients’ preferences and cultural backgrounds fosters a sense of ownership and empowerment, promoting a positive attitude toward oral health maintenance.

Customized Oral Hygiene Care Product Recommendations

Each patient presents with a different kind of oral manifestation, and a standard oral health care plan may be inadequate. Based on a thorough assessment and discussion, each product should be customized accordingly, depending on the patient’s status. The recommendations by oral health professionals can comprise different types of toothbrushes, toothpaste, mouthwash, and/or interdental cleaning aids [34]. All these dental cleaning aids facilitate the mechanical removal and disruption of dental plaque, but depending on the techniques and products used by the patients, they can be either effective or ineffective [35]. As a result, it is through recommendations and guidance by professionals that patients can decide which product suits them most. For the toothbrush, it is important to consider the suitability of the patient's oral hygiene method by looking at the handle, the size of the toothbrush heads, and the bristles, as well as the use of a powered or manual toothbrush [36].

Other types of toothbrushes, such as interdental brushes and compact-tufted brushes, should also be tailored according to the patient's needs. The use of interdental brushes is commonly emphasized by dental professionals to be used by patients with reduced periodontium, as this will facilitate the removal of interdental plaque that cannot be accessed by the normal toothbrush. However, the sizes of the interdental brushes used should be customized depending on the space of the interproximal area to allow optimum mechanical removal of dental plaque [35,37].

As for the dentifrices, their mechanism of action to aid in oral hygiene care depends on the active ingredients set by the manufacturers. Each active ingredient possesses different properties, such as an anti-caries agent by calcium chloride-based fluoride [38], an anti-calculus agent by pyrophosphate and zinc citrate [39], or a desensitizing agent by potassium nitrate and strontium chloride [40]. The same thing also applies to the need for the use of mouth rinses by patients, depending on ingredients such as chlorhexidine [41], chlorhexidine combined with fluoride [42], or essential oils [43]. These are only some of the examples of dentifrices and mouth rinses available from abundant other products that are available in the markets, depending on their active ingredients. By analyzing the patient’s needs and circumstances, oral health professionals should outweigh the risks and benefits of each product available.

Periodic Reassessment and Readjustment

After completion of the POHAM, patients should be regularly assessed regarding their oral health status, and any adjustments can be made based on changes in their medical status, psychosocial factors, and/or lifestyles [9]. A personalized plan should evolve with the patient's changing needs over time, and if there are any challenges encountered upon delivery of OHA, oral health professionals should review back on the POHAM and go through any missed steps and sequences that might be important in the success of OHA delivery.

## Conclusions

In summary, this review underlines the important shift towards personalized oral hygiene advice, emphasizing the need to tailor recommendations to individual patients. The proposed Personalized Oral Hygiene Advice Model (POHAM) is a novel approach that integrates comprehensive assessments while offering a dynamic approach for oral health professionals to use in the clinical setting. This model can be utilized as a guide to deliver OHA to patients and facilitate the improvement in patient engagement, adherence, and eventually overall oral health outcomes. As per current evidence, continued research, education, and technological advancements are crucial in realizing the potential of personalized strategies, fostering a more patient-centered, and effective oral health approach. 
